# Negative pressure wound therapy management of the “open abdomen” following trauma: a prospective study and systematic review

**DOI:** 10.1186/1749-7922-8-4

**Published:** 2013-01-10

**Authors:** Pradeep Navsaria, Andrew Nicol, Donald Hudson, John Cockwill, Jennifer Smith

**Affiliations:** 1 Department of Surgery, Groote Schuur Hospital, University of Cape Town, Cape Town, South Africa; 2 Smith & Nephew, St Petersberg, Florida, USA; 3 Smith & Nephew, 101 Hessle Road, Hull, HU3 2BN, UK

**Keywords:** Negative Pressure Wound Therapy (NPWT), Grade 1 and 2 open abdomen, Abdominal trauma, Fascial closure

## Abstract

**Introduction:**

The use of Negative Pressure Wound Therapy (NPWT) for temporary abdominal closure of open abdomen (OA) wounds is widely accepted. Published outcomes vary according to the specific nature and the aetiology that resulted in an OA. The aim of this study was to evaluate the effectiveness of a new NPWT system specifically used OA resulting from abdominal trauma.

**Methods:**

A prospective study on trauma patients requiring temporary abdominal closure (TAC) with grade 1or 2 OA was carried out. All patients were treated with NPWT (RENASYS AB Smith & Nephew) to achieve TAC. The primary outcome measure was time taken to achieve fascial closure and secondary outcomes were complications and mortality.

**Results:**

A total of 20 patients were included. Thirteen patients (65%) achieved fascial closure following a median treatment period of 3 days. Four patients (20%) died of causes unrelated to NPWT. Complications included fistula formation in one patient (5%) with spontaneous resolution during NPWT), bowel necrosis in a single patient (5%) and three cases of infection (15%). No fistulae were present at the end of NPWT.

**Conclusion:**

This new NPWT kit is safe and effective and results in a high rate of fascial closure and low complication rates in the severely injured trauma patient.

## Introduction

Management of the open abdomen is an area of medicine which has expanded rapidly over the last 20 years [1] and has resulted in decreased mortality rates [2]. The benefits of managing patients with open abdomens include prevention of intra-abdominal hypertension (IAH) and abdominal compartment syndrome (ACS), early identification of intra-abdominal complications (e.g. bowel ischemia) and ease of re-entry. Despite these benefits, maintenance of an open abdomen creates numerous management challenges such as development of fistula and infection. Prolonged maintenance of an open abdomen may also lead to a reduced chance of re-approximation of the fascia, as abdominal contents become ‘fixed’. With increasing adoption of open abdomen techniques has come an increased demand for Temporary Abdominal Closure (TAC) methods to protect the Open Abdomen during the phase of open treatment. Principal techniques for TAC are: Negative Pressure Wound Therapy (NPWT), Vacuum-Pac method (“Vac” Pac), artificial burr (Whitmann™ patch), absorbable mesh/sheet, zipper, “plastic silo”, skin closure and dynamic retention sutures. These techniques vary in their efficacy with regard to fascial closure rates, associated morbidity and mortality rates. A number of systematic reviews have concluded that the artificial burr and NPWT have the highest fascial closure and lowest mortality rates [3,4]. Because of its relative ease of application, and preservation of fascial tissue, NPWT is becoming a dominant choice for TAC in the open abdomen patient [1].

TAC can be appropriate in the treatment of OA derived from a wide range of traumatic, post-operative and septic clinical scenarios. Together these form a complex and diverse group of wounds. Much of the published literature describing outcomes in OA is difficult to interpret due to grouping together of these heterogeneous clinical scenarios with widely varying aetiologies, prognoses and even treatment goals. This leads to highly variable reported outcomes and complication rates. The rate of fascial closure in open abdomen patients treated with NPWT has been reported as low as 22% [5] (in pancreatitis) and as high as 92% [6] (in trauma). In order to understand how outcomes and potentially treatment protocols vary in different types of open abdomen patients, researchers must first publish results from homogenous and well-defined subgroups. The World Society of Abdominal Compartment Syndrome (WSACS) has proposed a simple clinical classification for describing the open abdomen (Bjorck et al.) [7] in order to facilitate comparison of study outcomes and clinical approach (see Table 1). The aim of the current study was to use the Bjorck classification to report outcomes of a well-defined group of patients, (with grade 1 or 2 open abdomens derived from traumatic injury) following treatment with a recently introduced NPWT system for TAC in the open abdomen. A systematic review of the literature, identifying studies with comparable homogenous study populations, was carried out as a means of comparing results from this study with results from the literature.

**Table 1:** Open abdomen classification

	
Grade 1A	Clean OA without adherence between bowel and abdominal wall or fixity of the abdominal wall (lateralization of the abdominal wall).
Grade 1B	Contaminated OA without adherence/fixity
Grade 2A	Clean OA developing adherence/fixity
Grade 2B	Contaminated OA developing adherence/fixity
Grade 3	OA complicated by fistula formation
Grade 4	Frozen OA with adherent bowel, unable to close surgically, with or without fistula

Adapted from Bjorck et al. [7].

## Methods

### Temporary abdominal closure

A prospective, open labelled, non-comparative study was carried out in two centres in South Africa between August 2010 and December 2011. Consecutive patients presenting with traumatic injury and 1) requiring damage control laparotomy with staged abdominal repair; or 2) developing abdominal compartment syndrome requiring laparotomy and temporary abdominal closure; or 3) with full thickness traumatic abdominal wall defects with exposed viscera requiring temporary abdominal closure were assessed for inclusion into the study. Patients with grade 1a,1b or 2a, 2b open abdomen, as classified by Bjorck et al. [7] (Table 1) were suitable for inclusion. The following exclusion criteria were also applied: <18 years, pregnant, malignancy in wound bed, unexplored fistulas, high risk for imminent death (as determined by the treating surgeon), pre-existing large ventral hernia, significant loss of abdominal wall fascia as a result of trauma or infection, patients with grade 4 open abdomen (Bjorck et al. classification, see Table 1), patients with a known history of poor compliance with medical treatment and any patients who had previously been withdrawn from the study. The trial was approved by local ethics boards at both institutions and was carried out in strict accordance with the Helsinki declaration. Informed consent was obtained where possible from the patient, but if the patient was incapable, the patient’s legal representative was asked to provide consent on the patient’s behalf. If this was not possible then independent physician consent was considered acceptable as approved by the local ethics committee. All patient information was anonymised at source.

Patients suitable for inclusion underwent initial damage control laparotomy, where initial control of haemorrhage and contamination was performed. This was followed by intra-peritoneal packing when required and TAC. Further resuscitation to near normal physiology in the intensive care unit (ICU) was continued. Re-laparotomy was performed at 48 hours or earlier if indicated. Negative pressure wound therapy (RENASYS-AB Abdominal Dressing and RENASYS EZ pump Smith & Nephew; St Petersburg, FL, USA) was applied to the wound in the following way. A fenestrated non adherent film was placed directly over the exposed viscera but under the rectus sheath. Polyurethane foam was then reduced along pre-cut perforations to the appropriate size and placed on top of the film within the open abdomen. A transparent film then covered the foam and the surrounding peri-wound skin before a suction port was connected to the NPWT pump. Negative pressure was delivered at a continuous -80 mmHg. The trial comprised a maximum of 20 days of treatment with the NPWT system with an additional 8 day post-treatment initiation follow up. Dressing changes usually took place at 48 hours during re-laparotomy for removal of packs and re-establishment of bowel continuity. Full medical and wound assessments were made. Wound closure was carried out when possible and at the discretion of the attending trauma surgeon.

The primary objective was to determine the number of days taken to achieve delayed primary fascial closure. Secondary objectives were mortality, change in OA classification, intra-abdominal pressure (IAP), length of stay (days) in ICU and hospital, incidence of complications (abdominal compartment syndrome (ACS), fistula formation, sepsis, multiple organ failure (MOF), acute respiratory distress syndrome (ARDS)). SOFA, APACHE, ISS, NISS scores were also recorded.

### Statistical evaluation

Kaplan-Meier estimate of the median time to achieve primary fascial closure by treatment discontinuation was presented. McNemar’s test was used to test for a reduction in the presence of infection from baseline to final assessment. All other outcomes were summarised using descriptive statistics.

### Systematic review

The PRISMA guidelines were used as a guide in designing the systematic review process [8]. The following PubMed search [("open abdomen" OR "abdominal compartment syndrome" OR laparotomy) AND ("negative pressure wound therapy" OR NPWT OR "Vacuum assisted" OR VAC OR "vac pack" OR "vacuum pack") NOT review] was carried out in April 2010 and updated in April 2011 and May 2012. These studies were reviewed manually and the following types were excluded: paediatric studies, studies where greater than 33% of patients had open abdomen wounds with advanced sepsis at baseline; Grade 4 wounds at baseline; Case reviews (fewer than 6 cases). Although the majority of studies did not classify the wounds according to Bjorck et al. [7], an attempt was made to classify them retrospectively based on the patient data provided. All studies carried out on non-septic Grade 1 or 2 open abdomen wounds were included regardless of aetiology. Raw data was extracted from all the papers. Outcomes (fascial closure, mortality and fistula) were expressed as a percentage of the total numbers of patients treated in order to minimise bias based on different sample sizes. This approach also corrected inherent reporting bias in several of the studies relating to whether data took numbers of deceased patients into account (i.e. expressed outcomes as a percentage of the entire cohort and not just percentage of survivors).

## Results

### Patients

Twenty trauma patients undergoing damage control laparotomy were recruited (see Table 2 for demographic and baseline wound details). Injury severity was measured by the Injury Severity Score (ISS) with a median value of 25 (range 9–50). An ISS of >15 (a measure of severe trauma) was present in 17/20 patients. Four (20%) patients died during the study period; One patient achieved primary fascial closure, but died following a cardiac arrest before the end of study period. Two other patients died as a result of acute renal failure and the remaining patient died as a result of multi-organ failure. Data for all 20 patients was included in all evaluations on an ‘intention to treat’ basis, unless specified.

**Table 2:** Patient and wound characterisation at baseline


Age; median (range)	31.4 years (22 – 44)
Male (% patients)	90%
BMI; median (range)	26.3 kg/m^2^ (17.7 – 50.8)
Injury Type (% patients)	
· Blunt trauma	50% (10/20)
· Penetrating Trauma	50% (10/20)
Injury scores (median (range)	
· SOFA	11 (0–17)
· APACHE II	14.5 (3–25)
· ISS	25 (9–50)
· NISS	33 (13–66)
IAP (# patients)	
· <12 mmHg	10
· >12 mmHg (IAH)	10

IAP = intra-abdominal pressure; IAH = intra-abdominal hypertension as defined by Cheatham et al. 2007 [9].

### Primary objective - fascial closure rate

Fascial closure was achieved in 13 out of 20 patients (65% of patients on an intent-to-treat basis) (see Table 3; see supplemental data for Kaplan-Meier estimate data). Fascial closure rate expressed as the percentage of survivors was 75% (12/16 patients) (data not shown). One patient died following fascial closure but the remaining 12 closed abdomens were stable at a follow up 8 days after closure although a superficial wound sepsis was present in one. The median time to achieve primary fascial closure was 3 days (CI) (n=20). Two patients were withdrawn from the study after 19 and 24 days of NPWT therapy because they developed a Grade 4 (fixed) abdomen and fascial closure was no longer an option (i.e. they could no longer contribute to the primary objective). Each open abdomen was graded according to the WSACS classification [7] (Table 1) at the initial application of NPWT and at each subsequent dressing change, including the final removal of the dressing. The grade of open abdomen for the majority of patients improved during the course of therapy.

**Table 3:** Progression of open abdominal wounds from initial presentation to end of therapy

**Grade**	**Baseline**	**End of therapy**
Closed	0	13 (65%)
1a	14 (70.0%)	2 (10%)
1b	5 (25.0%)	1 (5%)
2	1 (5.0%)	2 (10%)
2c	0	0
3	0	0
4	0	2 (10%)
N	20 (100%)	20 (100%)*

Progress of the wounds during therapy was assessed using the Bjorck et al. classification system. *one patient died less than 24 hours after having a baseline assessment. As no other data was available, it was assumed that the wound grade at death was the same as the baseline assessment (Grade 1A).

### Secondary objectives

SOFA and APACHE11 scores decreased from medians of 11 and 14.5 at baseline to 9 and 12 respectively at the end of therapy. There was no apparent relationship between IAP at baseline and achievement of fascial closure. Median time in ICU was 8 days (range 1–28 days, n=20). In the remaining patients, reasons for discontinuation of NPWT were death, (3/20; 15%), poor compliance (1/20; 5%), withdrawal for other reasons (1/20; 5% - persistent bowel hematic as a consequence of an extremely large viscera). Fluid contained in the waste canister was approximately measured and this formed part of the daily fluid management of the patient. A mean volume of 871 ml (median 700 ml) was present in the canister at dressing change. Blood loss into the canister was also an early sign of internal bleeding and allowed rapid intervention (data not shown).

A range of complications were assessed and results are shown in Table 4. One fistula (5%) was observed during the study in a single patient who had received penetrating trauma. This low output fistula was observed during the second dressing change but had resolved by the next dressing change (48 hours later). No trauma was observed on removal of any of the dressing components and was therefore unlikely that adhesion of the dressing to the bowel had contributed towards the fistula formation.

**Table 4:** Number of patients developing abdominal wound related complications

	**Incidence**		
**Complication**	**Baseline**	End of therapy*	**At any point during therapy**
Fistula	0	0	1 (5%)
Bowel necrosis	1 (5%)	1 (5.3%)	2 (10%)
Bowel evisceration	4 (20%)	2 (10.5%)	5 (25%)
Infection / sepsis	5 (25%)	5 (26.3%)	8 (40%)

The incidence of complications was recorded per patient. N=20 except * (where n=19 due to one patient dying after having a baseline assessment).

Bowel necrosis was found in two patients (10%). One instance was present at baseline and was resolved prior to application of NPWT following surgical removal of 90 cm length of bowel. This patient went on to achieve fascial closure within 3 days of injury. The second instance of bowel necrosis developed at the second dressing change during the study in a patient who had a septic abdomen at baseline with a moderate degree of oedema. This patient died as a result of multi-organ failure due to sepsis and as a result of late presentation. The development of bowel necrosis was not believed to be related to the use of the NPWT device.

At baseline assessment, 5 patients had severe contamination of the abdominal cavity due to intestinal spillage. In 3 patients the contamination was controlled and there were no sign of contamination or infection by treatment discontinuation. The remaining 2 patients developed a clinically infected wound along with a further 3 patients during the course of the study. One patient, despite fistula resolution (as described above), became persistently infected preventing wound closure. The wound degraded into a grade 4 (fixed) open abdomen and was closed with a graft. A second patient with a grade 1a abdomen was progressing well but became confused and removed the dressing resulting in wound infection and withdrawal of the patient for non-compliance. The third patient who developed infection also developed bowel oedema throughout the study and evisceration. This was in part due to unusually large viscera. Therefore, at treatment discontinuation 5 patients’ abdominal wounds were clinically infected.

### Case study

**Figure 1:**
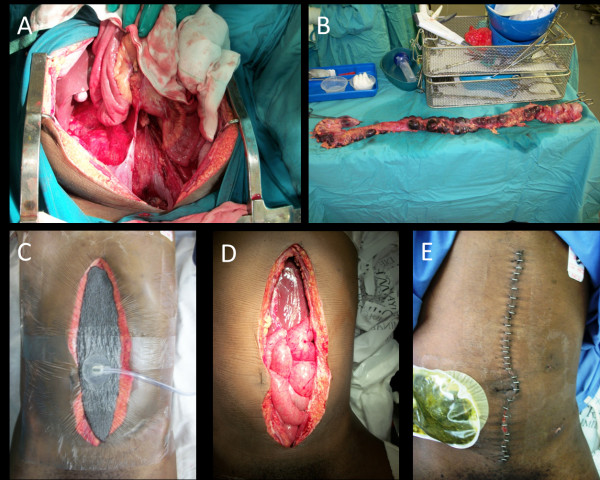
**A 27 year old male was admitted with blunt abdominal trauma.** A damage control laparotomy was performed **(A)**, 90 cm of necrotic bowel removed **(B)** and NPWT (Renasys F-AB, Smith & Nephew) applied at -80 mmHg **(C)**. Second look lapartomies were performed at 24 and 48 hours **(D)** and the fascia closed at Day 3 post injury **(E)**.

A 27 year old male with no significant medical history was admitted 18th October, 2010 with blunt trauma to the abdomen as a result of assault. A midline laparotomy for damage control was performed (Figure 1A). Severe contamination of the peritoneal cavity due to hollow viscous injury were apparent. Intra-abdominal pressure (IAP) was 15 mmHg and abdominal perfusion pressure (APP) was 58 mmHg. Injury scores were as follows: SOFA 11, APACHE 5, ISS 25 and NISS 48. The wound was classified as a grade 1b and was complicated by the presence of necrotic bowel. Ninety centimetres of bowel were removed surgically (Figure 1B) before the NPWT dressing was applied (Figure 1C) with the intention of performing a second look laparotomy to ensure no progression of bowel necrosis. NPWT pressure was applied at -80 mmHg continuous pressure. 800 ml of ascites was removed. Active resuscitation for 24 hours was required at which point a re-laparotomy was performed in order to view the rectal stump and rigid sigmoidoscopy. A second re-laparotomy was required at 48 hours (Figure 1D). The abdomen was closed by delayed primary fascial closure on Day 3 (Figure 1E) with no further complications.

### Comparison with published literature

In order to compare the results presented here with the existing literature, a systematic search was carried out. Table 5 shows the process of the systematic search. Briefly 129 papers were identified, of which 49 passed the selection criteria and were appropriate for detailed review. Of these, a further 13 did not report relevant end-points. Of the remaining 36 papers, studies where >33% of the study population was septic were excluded because the presence of sepsis has a significant effect on the prognosis and outcomes of the open abdomen patient [10]. In the present study, 25% of wounds at baseline were infected or contaminated. Studies using ‘home-made’ NPWT systems (i.e. vac-pack) were excluded to avoid any variability in outcomes resulting from variability in components or technique of application. Vac-pack has also been reported to have slightly less effective outcomes compared to VAC [4,11] therefore commercial NPWT provided a good benchmark. Open abdomen wounds from all aetiologies were theoretically included but in practice the majority of studies reported traumatic patients with only 2 studies reporting mixed cohorts of patients.

**Table 5:** Systematic review chart

**Total number of papers identified**		**129**
Reason for exclusion	Duplications	4
	In vivo studies	9
	Paediatric	4
	Significant modification to application technique	14
	Irrelevant clinical area	21
	Reviews/comments/letters	9
	Case series <6	18
**Number of papers reviewed**		**48**
Reason for exclusion	No relevant endpoints	13
	Vac-pack removed *	13
	Cohorts with >33% septic	15
**Number of remaining papers**		**8**

*papers describing results with a non-commercial NPWT technique known as ‘vac-pack’ were excluded.

Results of the comparison between the present study and relevant articles identified from the systematic review are shown in Table 6. The identified studies are relatively small in size with a mean patient number of 30. Demographic variables (ISS, age, gender) were acceptably similar between this study and the reported studies (data not shown). Overall, mean fascial closure rates of 63.7% were reported; a close match with the mean value of 65% reported in the current study. These values reflect the ‘intent-to-treat’ population which includes all patients regardless of whether they survived their injuries. Mean mortality rate in the published studies was 22% which compares well with the values in the current study of 20%. A 3% mean percentage of patients in the published literature developed a fistula during therapy (ranging from 7 to 0%). The value in the current study of 5% compares well, especially considering that a single patient developed a fistula which was apparent at only one dressing change and was resolved by the next dressing change. In terms of the rate of other complications, the data was less reliable because not all the relevant studies reported complications (not shown). In conclusion, there is no evidence that the device used in this study is any less efficacious than the VAC™ device in the treatment of Grade 1 and 2 open abdomen wounds derived from traumatic patients.

**Table 6:** Comparison with published literature

**Reference**	**Method**	**n**	**Fascial closure**	**Mortality**	**Fistula**
**This Study**	**RENASYS -AB**	**20**	**13 (65%)**	**4 (20%)**	**1 (5%)**
Miller et al. 2004 [12]	VAC™	53	38	8 (15%)	1 (2%)
Garner et al. 2003 [6]		14	13	NR	0
Suliberk et al. 2003 [13]		29	25	6 (21%)	2 (8)
Stone et al. 2004 [14]		48	23	16 (33%)	2 (4%)
Weinberg et al. 2008 [15]		9*	6	NR	NR
Arigon et al. 2008^†^[16]		22	6	3 (14%)	0
Batacchi et al. 2010 [17]		35*	NR	8 (23%)	NR
Labler et al. 2005 [18]		18	12	5 (33%)	0
**Total patients reporting relevant end-point**		**228**	**193**	**205**	**5**
**Weighted mean (%)**			**63.7**	**23.5**	**2.7**

NR = Not Recorded. NA = Not Applicable. * refers to the relevant subgroup (treated with NPWT) of a wider analysis. ^†^ data extracted from abstract only (article in French). All studies described traumatic patients except Arigon et al. [16] and Batacchi et al. [17] who described a mixed group of aetiologies with the majority of reported patients being relevant to this study.

## Discussion

In this study, the rate of fascial closure was 65% on an intent-to-treat basis which compares well with comparable published studies (63.7%) of patients (Table 6). All comparisons were carried out with studies using the predominant commercially available abdominal NPWT kit, Abdominal VAC™ (KCI San Antonio, Tx USA). One significant drawback of this study design was the non-comparative design. A large comparative study would be required to confirm equivalence of these two devices. The present study provides evidence that application of the alternative dressing (RENASYS™ AB Smith & Nephew St Petersburg, FL USA) is likely to achieve similar outcomes. Concurrent application of fascial tension: for example through the use of ‘dynamic suturing’, along with NPWT may further improve the frequency of fascial closure [19,20] although, to date, no comparative studies have been carried out to support this. Achievement of fascial closure not only has significant implications for the recovery of the patients but also leads to shorter ICU and hospital length of stay, reduced need for surgical reconstruction of the abdominal wall, and shorter recovery time. These factors all have a considerable cost element so early but safe abdominal closure is the best outcome.

The most commonly cited objection to the use of NPWT TAC is a perceived increase in fistula formation. The rate of fistula formation in the current study of 5% was similar to that derived from the published studies of 3%. It is possible that these relatively low levels of fistula formation are observed in this specific population of open abdomen patients [2,21] and that higher incidence of *de novo* fistula formation may occur in ‘high risk’ subsets of patients i.e. those with more advanced grade of open abdomen (grade 3 or 4), sepsis, or in wounds where a bowel anastomosis following bowel surgery is present or where there is a delay or failure to achieve fascial closure. In fact where concern has been expressed by several commentators [22-24] the patients described tend to be ‘high risk’. The potential link between NPWT and fistula formation has been disputed by others [25] including in a systematic review [26]. More evidence is needed to determine whether use of NPWT on grade 3 or 4 open abdomen is effective and whether an increased risk of fistulisation is indeed observed as a result of therapy in this sub-population. With regard to the current study, one drawback is the relatively low sample size, which may not accurately reflect the true incidence of fistula formation in these wounds. One variable not assessed in the systematic review was the level of negative pressure used in each study. This is reported in only one study where the relatively high level of -175 mmHg was used [13]. Use of high levels of negative pressure is thought to a potential risk factor for increased fistula formation but the present analysis is not able to clarify this assertion.

Wider adoption of the published classification system is needed when reporting outcomes on open abdomen patients in order to help clarify these and other issues.

## Conclusion

Application of an alternative NPWT TAC system, when applied to trauma patients with grade 1 and 2 open abdomens (Bjorck et al. classification) [7] is safe and effective resulting in a high rate of fascial closure rate (65% intent-to-treat) and relatively low rate of complications. These values are similar to those presented in the published literature. Wider adoption of the published classification system is needed when reporting outcomes on open abdomen patients.

## Abbreviations

NPWT: Negative Pressure Wound Therapy; IAH: Intra-abdominal Hypertension; IAP: Intra-abdominal Pressure; ACS: Abdominal Compartment Syndrome; OA: Open Abdomen; SOFA: Sequential Organ Failure Score; APACHE 11: Acute Physical and Chronic Health Evaluation Score; ISS: Injury Severity Score; NISS: New Injury Severity Score.

## Competing interests

This study was funded by Smith & Nephew (S&N). Authors JS and JC are employees of S&N. DH was part of an International Expert Panel on Negative Pressure Wound Therapy funded by an unrestricted educational grant provided by Smith & Nephew.

## Authors’ contributions

PN, DH and AN acquired the data. JC and AN conceived and designed the study and JS interpreted the data, drafted the manuscript and carried out the systematic review. All authors provided critical revisions of the manuscript before their final approval of the manuscript.
